# A spatial econometric analysis of the environment Kuznets curve and pollution haven hypothesis in Sub-Saharan Africa

**DOI:** 10.1007/s11356-023-26306-9

**Published:** 2023-03-28

**Authors:** Jamiil Jeetoo, Earnest Rungano Chinyanga

**Affiliations:** 1 Department of Accounting, Finance and Economics, School of Business, Management and Finance, University of Technology, Mauritius, Pointes-Aux-Sables, Port Louis, Mauritius; 2 Centre for Competition, Regulation and Economic Development, College of Business and Economics, University of Johannesburg, Johannesburg, South Africa; 3 Department of Economics, University of Zimbabwe, Harare, Zimbabwe

**Keywords:** CO_2_ emission, Environment Kuznets curve, Natural resource depletion, Pollution haven hypothesis, Spatial interactions

## Abstract

The aim of this study is to test the environment Kuznets curve (EKC) and pollution haven (PH) hypotheses in Sub-Saharan Africa (SSA). An important methodological point that has been over-looked by many studies is that environmental quality is not only correlated in time but also in space. For this purpose, the study applies spatial panel econometric analysis using a balanced panel of 35 SSA nations from 2002 to 2015 to examine the EKC and PH hypotheses. Both spatial interdependence and individual heterogeneity are accounted for through the application of the spatial Durbin model (SDM) so as to avoid potential bias and inefficiencies in parameter estimates. As proxies for environmental quality, panel data aggregates on carbon dioxide (CO_2_) emissions and the depletion of natural resources are utilised. The findings offer proof for the EKC theory about the depletion of natural resources in SSA. The EKC theory, however, does not apply to CO_2_ emissions. Moreover, the study finds that the positive scale effect of trade outweighs the negative technique effect of trade, which indicates that trade liberalisation has a negative effect on both environmental quality indices. This discovery supports the PH theory. The study also demonstrates positive spatial spill-over for natural resource depletion between neighbouring countries and negative spatial spill-over for carbon dioxide emission between close countries.

## Introduction

A fundamental issue that academics and politicians have had to deal with throughout the years is achieving sustainable development (Tachega et al. 2021). National economies are quickly integrating into a global one as international economic links get stronger. As a result, the link between economic growth and environmental sustainability is a topic of constant discussion. Sustainable Development Goals (SDGs) place a high priority on environmental conservation because it significantly affects both climate change and global warming (United Nations 2014; United Nations 2021). Thus, it is vital to strike a balance between environmental sustainability and growth. It is crucial to recognise that African nations are transitioning from being more agrarian to industrialised nations, and this raises concerns about Africa’s role in environmental degradation (Tachega et al. 2021).

Examining the consequences of globalisation on environmental quality is wise as nations become more industrialised. According to the EKC theory, environmental degradation grows with economic expansion initially until it meets a specific turning point, which is followed by an improvement in environmental quality as income increases (Shahbaz et al. 2016). African countries are amongst the least explored or investigated countries for the EKC (Shahbaz et al. 2016). This is because the continent emits considerably fewer CO_2_ emissions than other continents (Tachega et al. 2021). Africa currently provides only 4% of global CO_2_ emissions (Ayompe et al. 2021). However, these nations face dangers from climate change as a result of rising industrialisation. The rate at which African nations are growing may indicate that the continent’s emissions will continue to rise, with some generating CO_2_ emissions at levels comparable to those of the industrialized world. Furthermore, the EKC hypothesis emphasizes the importance of formal responses to environmental degradation based on government regulations, which are typically more aggressive in wealthier economic systems. While stating that geographical heterogeneity in the environment-income link may reveal additional objectives for integrated socio-environmental policy, there is a dearth of research regarding the significance of space in the EKC framework (Mosconi et al. 2020).

Additionally, the transfer of industries from industrialized to poor countries has been made easier by greater globalisation. As a result, the EKC modelling and the justification of the connection between income growth and environmental quality both require an understanding of international trade. Furthermore, free trade encourages growth that is conducive to pollution-intensive sectors that devastate regional surroundings because of inadequate environmental regulations. This is a result of the shifting of production that produces large amounts of pollution from nations with strict environmental rules to those with laxer laws. The pollution haven (PH) hypothesis is the name given to this circumstance (Levinson and Taylor 2008). Therefore, it is essential to research whether African nations have evolved into pollution hotspots over time.

Numerous panel econometric models are used in the literature to test the EKC and PH hypotheses. However, the majority of these researches concentrated on the temporal aspect, ignoring the spatial component. A limitation of these approaches is that they ignore the impacts of spatial correlation amongst the cross-sections within a panel of countries (Ancelin and LeGalloH 2008). In particular, with globalisation and growing cross-country connections, research have relied on the notion that nations evolve as distinct entities which is not always practical (Elhorst et al. 2013; Jorge Chica-Olmo et al. 2020). The failure to include pertinent spatial spill-over factors in econometrics analysis can be a serious methodological flaw that results in inaccurate and ineffective estimates (LeSage and Pace 2010; Elhorst 2010). A spatial econometric model can accommodate for spatial correlations amongst the cross-sections and account for the time-specific effects (Elhorst 2017). In addition spatial regressions are also robust in handling spatial heterogeneity in data analysis (Srinivasan 2015). This is important as spatial dependence can occur from both homogeneous and heterogeneous spatial properties within a group of spatially dependent countries or regions (Murshed et al. 2020).

In the SSA context, it is vital to consider the existence of spatial effects in modelling environmental quality. This can be due to different reasons. Various regional policy tools have been employed at international and regional level environmental quality. Furthermore, cross-country spatial interaction is related to Tobler’s first law of geography stating that “everything is related to everything else, but near things are more related than distant things” (Tobler 1970). As such, no region should be assessed under the assumption that they are spatially independent and isolated. In fact, countries in SSA are very much connected given that they share common geographical and climatic conditions (Espoir and Sunge 2021) and nearby countries belong to similar regional blocs. The latter, coupled with globalisation and policies regarding trade liberalisation and economic integration in SSA, reinforces the connection and likely spill-over effects across countries in SSA. Moreover, the concept of spatial spill-over has been included in many studies in SSA such as in trade liberalisation, economic integration, and globalisation mean stronger ties amongst countries. As such, there is a greater likelihood of economic shock spill-overs across the continent due to the phenomenon of “yardstick competition” where governments in SSA may tend to match the policies of close countries or due to demonstration effects, where some governments may follow the policies of other nearby countries (Jeetoo 2022a, b).

In addition, developing countries are often involved in intense competition with their neighbours to promote trade and investment with the developed world. Thus, investment and trade activities of neighbouring countries can have spill-overs which could play an important role in shaping the EKC. Developing countries might relax their environmental policies to promote trading relationships with developed countries (Ulph 1992). Consequently, trade may transport pollution from high- to low-income countries. Therefore, there is the possibility of spatial dependency of pollution emissions in the SSA region. In the presence of statistically significant spatial dependency, the estimation of non-spatial models can result in mispecification bias and thus leading to misleading results (Anselin 1988). As a result, there is a possibility that environmental degradation will be spatially dependent throughout the Africa (Mahmood et al. 2020b).

Although there are rules in place to address environmental degradation, emerging nations in SSA are increasingly placing a higher priority on growth and trade than environmental quality. As a result, the study uses the SSA nations to examine the EKC hypothesis while applying spatial analysis. It also focuses on testing the PH hypothesis in these nations. This work contributes to the current body of literature in multiple ways which were not part of the few studies done on the SSA countries. First, the study adds the spatial analysis component to the EKC model in SSA countries being the first study of its kind to consider spatial interactions in examining the EKC in the SSA context.

Appraising the effect of spatial spill-overs in the context of environmental quality would improve understanding of the interactions across SSA. For example, results on whether neighbouring countries’ environmental quality pattern can influence a country’s environmental quality can bolster policymakers’ efforts in coordinating regional interventions. Moreover, given the scarce evidence on the role played by trade liberalisation on environmental quality, this study contributes by testing the PH hypothesis. In addition, the study also incorporates a sustainability indicator of environmental quality which is natural resources depletion. This will help to ascertain whether the SSA region is on a sustainable development path or not and is an alternative environmental quality indicator not tested under the EKC framework in SSA.

The remainder of this paper is structured as follows. A summary of the theoretical and empirical literature is presented in the “Literature review” section. A description of the data is found in the “Methodology and data” section. The methodology is presented in the “Methodology and data” section, which also introduces the spatial econometric framework. The “Empirical results and discussion” section presents and discusses the results. The conclusion and final discussion of policy implications are included in the “Conclusion and policy implications” section.

## Literature review

### Theoretical review

#### The EKC theory

The EKC hypothesis is the notion explaining the connection between growth and environmental deterioration (Dinda 2004). The EKC follows Kuznets (1955), who claimed the existence of an inverted U-shaped link between income disparity and economic growth. The EKC and the traditional Kuznets curve are predicated on the notion that as the economy expands, some indicators of quality of life, such as income inequality and environmental quality, get worse before getting better. As a result, Grossman and Krueger (1991), who initially proposed an inverted U-shaped connection between environmental degradation and economic growth, are to be given credit for the concept underpinning the EKC model. Accordingly, environmental deterioration (CO_2_ emissions) first rises with an increase in per capita income until it reaches a certain point, after which it begins to decline (Tachega et al. 2021; Shahbaz et al. 2016; Adu and Denkyirah 2017). At the initial stage of development, there is little concern for environmental issues, with little to no environmental consciousness, and no environmentally beneficial technologies. Beyond a certain point, the environmental quality starts to improve. People will start to want a better environment so long that per capita income keeps rising beyond the threshold level; thus, they will start working to make the environment better.

Globalisation and international trade are important factors in explaining the EKC in the same context (Dinda 2004). Three components are mentioned by Grossman and Krueger (1991, 1995) in their discussion of the growth-environment nexus. These are the composition effect, the technique effect, and the scale effect. First, according to the scale effect, as economic growth rises, current environmental issues only get worse. This is due to the fact that in the early phases of expansion, a lot of inputs, such as natural resources (energy, land, etc.), are needed to produce more (Antweiler et al. 2001 and Wasiu and Alasinrin 2015). As a result, these inputs result in more production, which means higher by-products such as trash and pollution (Dinda 2004). This indicates that when the economy grows, the environment suffers due to the scale effect. As a result, the scale effect intensifies the level of regional and global pollutants like carbon dioxide emissions as well as the depletion of natural resources.

The technique effect explains why environmental quality improves when revenue rises. As income rises, there is a trend for environmental quality to improve. Therefore, this impact considers the environment to be a typical good. The demand for a typical good rises as income does. This implies that as money rises, environmental concern also does. As a result, it is conceivable for a high-income nation to implement strict environmental policies. As a result, a high-income nation can invest heavily in research and development and buy pertinent technology that lowers emissions and safeguards the environment.

Countries typically shift to businesses that produce less pollution due to the composition effect on growth, which modifies the structure of economic activities (Wasiu and Alasinrin 2015). Therefore, an economy that is able to shift production towards items that require a lot of natural capital pollutes more, maintaining the impacts of scale and technique constant. Countries that abandon businesses reliant on natural resources produce less pollution. Due to their reliance on natural resources, developing nations in the SSA region are likely to specialise in resource-intensive production, which will exacerbate the depletion of those resources and increase pollution.

However, the EKC hypothesis remains debatable (Dinda 2004). In particular, there are different findings from the literature regarding the EKC’s turning points. Due to the fact that the EKC differs depending on the type of pollution, researchers do not have a generally acknowledged shape for it. Therefore, Dinda (2004) summarises this by saying that “…there is no agreement in the literature about the income level (turning point) at which environmental degradation starts to improve.”

#### PH hypothesis

According to the PH hypothesis, countries’ environmental regulations have an impact on where industries choose to locate. Varying nations have different compliance costs. Therefore, one may anticipate that firms that produce high levels of pollution might move to nations with cheap compliance costs (Kirkpatrick and Scrieciu 2008; Millimet and Roy 2016). Heavy polluting industries might shift some industrial activities to nations with laxer environmental regulations thanks to globalisation and commerce, which changes the geographic distribution of economic activity and trade patterns by fostering havens for polluting companies (Millimet and Roy 2016). As a result, nations with lax environmental restrictions draw more polluting companies to locate their factories, giving them the freedom to emit more emissions and other externalities. Due to their constant demand for economic expansion and lack of environmental awareness, people in developing nations have fewer environmental restrictions, which attracts more businesses that produce dirt/polluting goods (Li et al. 2022).

### Overview and extension of empirical review

There is an immense and continuously growing literature on the EKC hypothesis since the seminal research of Selden and Song (1994), Grossman and Krueger (1995), and Panayotou (1997), in numerous countries and regions. The recent additions to the literature feature advancement in modelling techniques and the availability of richer datasets. As such, this review of the literature does not cover the whole EKC literature (for more details see Dinda (2004); Stern (2004); Chowdhury and Moran (2012); Shahbaz et al. (2016); Bah et al. (2020)) but focuses on the extension of this study to the empirical literature.

Bah et al. (2020) provide a systematic review of the studies regarding the SSA region. Amongst the numerous studies, different results are found using different techniques and datasets, with the majority showing evidence of the EKC effect in SSA (Kivyiro and Arminen 2014; Osabuohien et al. 2014; Gao and Zhang 2014; Shahbaz et al. 2016; Oshin and Ogundipe 2015; Lin et al. 2016; Zoundi 2017; Ouoba 2017; Dong et al. 2017, 2018; Wang and Dong 2019), while Omojolaibi (2010) and Ben Jebli et al. (2015) find the contrary. For instance, Ben Jebli et al. (2015) use the panel full modified ordinary least square (FMOLS), controlling for GDP per capita, renewable energy and trade using a panel of 24 SSA countries over the period 1980–2010, but find no evidence of the EKC, while Oshin and Ogundipe (2015) have applied the fixed effect (FE) and random effect (RE) methods, controlling for GDP per capita, literacy rate, population density, trade, and institutional quality using a panel of 15 West African countries over the period 1980–2012 and find evidence of the EKC. Differences may have arisen following the application of diverse econometric methods and sample selection and omitted variable bias issues (Raheem and Yusuf 2015).

Furthermore, Shahbaz et al. (2016) incorporate globalisation and energy intensity into the CO_2_ equation to examine the existence of the EKC in 19 African countries and uses the autoregressive distributed lag (ARDL) bounds test approach for cointegration. The findings reported that energy intensity positively influences CO_2_ emissions in South Africa, Cameroon, Congo Republic, Ghana, Libya, Nigeria, Morocco, Kenya, Sudan, Togo, Algeria, Angola, Africa, and Tunisia. Energy intensity has a negative effect on CO_2_ emissions in Zimbabwe and Zambia. The EKC model is found in Africa, Algeria, Congo Republic, Morocco, Cameroon, Zambia, and Tunisia.

Dong et al. (2017) explore the EKC over the period 1985–2016 sample of BRICS countries and conducted the panel unit root, cointegration, and causality tests allowing for cross-sectional dependence. The study employs the augmented mean group (AMG) estimator and concluded that there is strong evidence in favour of the EKC hypothesis for the BRICS countries. Using a similar methodology, Dong et al. (2017) and Wang and Dong (2019) find similar results in 13 Asia–Pacific countries over the period 1970–2016, and in 14 SSA countries over the period 1990–2014, respectively.

Ogundari et al. (2017) examine the EKC model SSA region focusing on deforestation and all greenhouse has (GHG) emissions for the period 1990–2009. The study uses a panel for 43 SSA countries. The findings from the feasible generalized least square (FGLS) indicate that the EKC model is present in the case of GHG from agriculture and it was not present in the case of deforestation. The study further finds that agriculture production and trade openness are positively related with all environmental indicators. Population growth is found to have a positive relationship with deforestation. In contrast to findings by Ogundari et al. (2017), Demissew Beyene and Kotosz (2020) use data for 12 East African countries and apply the pooled mean group (PMG) method to test the EKC and uncover a bell-shaped relationship between CO_2_ emissions and per capita income.

Sharif et al. (2019) apply the second-generation econometrics of panel data to analyse the cross-section independence and control the heterogeneity between cross-sections. The CIPS unit root test, Westerlund (2007) bootstrap cointegration, Pedroni cointegration, FMOLS, and heterogeneous panel causality techniques have been applied. The results affirm that all variables are integrated over the long run. The results also show that the nonrenewable energy consumption has a positive effect on environmental degradation, while renewable energy has a negative impact on environmental degradation and helps to reduce environmental hazards. Likewise, financial development also has a negative and significant impact on environmental degradation. Further, the Kuznets hypothesis is also tested and its existence is confirmed.

Andree et al. (2019) explore the relationship between economic growth in per capita GDP and carbon emissions using data between 1990 and 2014. The study reveals a U-shaped correlation between the per capita GDP and environmental degradation factors. It makes use of machine learning techniques to establish a relationship between the given variables across economic factors.

Sharif et al. (2020b) explores the impact of renewable and nonrenewable energy consumption on Turkey’s ecological footprint. The study applies the quantile autoregressive lagged (QARDL) approach for the period of 1965Q1–2017Q4. It further applies Granger-causality in quantiles to check the causal relationship amongst the variables. The results of QARDL show that the error correction parameter is statistically significant with the expected negative sign for all quantiles, confirming the existence of significant reversion to the long-term equilibrium connection between the related variables and ecological footprint in Turkey. In particular, the outcomes suggest that renewable energy decreases ecological footprint in the long run on each quantile. Furthermore, the results of QARDL confirm the EKC in Turkey. Sharif et al. (2020c) and Suki et al. (2020) also use the quantile autoregressive distributed lag (QARDL) model to test the environmental Kuznets curve, and the outcomes confirm the presence of the inverted U-shaped curve in the Malaysian economy.

Wan et al. (2022) investigate the role of natural resources, green financing, and environmental regulations in reducing carbon emissions in China using data from 2000 to 2019. The study uses the Bootstrap ARDL proposed by McNown et al. (2018). The latter method covers advanced cointegration features in the dynamic process. The long-run estimations of Bootstrap ARDL show that natural resource management, green investment, and environmental taxes abate environmental emissions.

Jian and Afshan (2022) examine the nexus between GFIN, GTIs, and carbon neutrality in G10 economies. The study employs a CS-ARDL estimator to analyse long-run and short-run relationships amongst model variables considering the data’s cross-sectional dependency, slope heterogeneity, unit root, and cointegration properties. The results confirm the presence of cross-sectional dependence, heterogeneity in the slope coefficients, stationarity properties, and cointegration between the variables of interest. It finds that economic growth tends to increase carbon emission at an early stage, but after a certain threshold, higher income reduces the ecological burden. The study validates the EKC hypothesis.

Deng et al. (2022) empirically examine the impact of financial inflow on renewable energy consumption and environmental quality in BRICS using data from 1991 to 2019. The basic results of the study emanate from the NARDL-PMG, but robustness is observed through FMOLS and DOLS. A positive change in FDI has a positive effect on CO_2_ emissions, whereas a negative change in FDI significantly reduces CO_2_ emissions in the long run. Moreover, positive and negative shocks to remittance lead to higher renewable energy consumption in the long run.

Sharif et al. (2022) employ advanced panel econometric modelling to address panel data analysis concerns, such as cross-sectional dependence, structural break, and slope heterogeneity (the Banerjee and Carrion-iSilvestre unit root and cointegration test and cross-sectional augmented ARDL). The study shows that green technology innovation together with green financing have a negative but significant impact on CO_2_ emissions. Whilst economic growth has shown a positive and significant impact on CO_2_ emissions in the G7 countries, social globalisation positively moderates the relationship between CO_2_ emissions and GDP, but negatively and significantly causes green financing and green technology innovation with CO_2_ emissions amongst the G7 countries.

More recently, spatial interaction effects have also been included in the EKC framework for various reasons. First, countries tend to follow and imitate the policies of neighbouring countries (Maddison 2006). In this sense, countries can mimic the environmental policies of close countries and thus affecting their environmental policies and conditions (You and Lv 2018). Second, spatial interaction is an important part of environmental issues, explaining the process by which air pollution is diffused and water contamination is spread from one geographic region to another (Wang et al. 2013). Excluding these spatial features in the EKC analysis can result in biasness and inefficient parameter estimates (Hao et al. 2018).

Regarding spatial modelling of the EKC model, Chuai et al. (2012) examine the altering trends of the spatial pattern at the geographic level of CO_2_ emissions from energy consumption, spatial autocorrelation assessment of CO_2_ emissions, spatial regression between CO_2_ emissions, and the explanatory variables. The findings indicate that the spatial effect of high as well as low emission regions does not alter although CO_2_ emissions increased by above 148% between 1997 and 2009. Also, the spatial regression analysis indicates that CO_2_ emissions are closely related to GDP and population.

Wang et al. (2013) estimate the EKC for ecological footprint at the global scale using a spatial econometric model. According to the study, there is proof of an EKC with an inverted U shape. The ecological footprint of consumption and production, income, and neighbouring countries’ biocapacity all have an impact on the domestic ecological footprint. The study also reveals that the production footprint is more susceptible to biocapacity, whereas the consumption footprint is more sensitive to local income.

Hao et al. (2016) use spatial econometric approaches to test the EKC hypothesis for per capita coal consumption in China. The study controls for spatial dependency and finds that there exist spatial correlations in coal consumption across the provinces in China. It finds that the EKC hypothesis holds between per capita coal consumption and GDP per capita. Kang et al. (2016) examine the EKC for CO_2_ emissions in China using spatial econometric modelling over the period 1997 to 2012. The study compares the turning points using the spatial panel model and non-spatial panel models. It shows that there is an inverted N-trajectory relationship between economic growth and CO_2_ emissions. The shape of the EKC is found to have been affected by the spatial spill-over effects. The study also finds emissions to be increased by urbanisation and coal combustion.

Wang and Ye (2017) re-examined the EKC for China’s city-level CO_2_ emissions. The study uses city-level data and a spatial econometric approach. The findings reveal that there is an uneven landscape of CO_2_ emissions at both provincial and city scales. Balado-Naves et al. (2018) examine to what extent per capita income carbon dioxide emissions are influenced by energy consumption, energy intensity, real per capita income, and the share of the service sector in GDP through considering the spatial relationships. The study comprises panel data for 173 countries for the period 199–2014. The study estimates the EKC model which is augmented by neighbouring per capita income and energy intensity. The study finds an inverted U-shaped EKC between neighbouring per capita income and national emissions per capita in Europe, Asia, and the whole world. The study also reports a positive relationship between neighbouring energy intensity and national emissions per capita.

A study by Hove and Tursoy (2019) investigates the EKC model in emerging economies. The study uses the generalized means model (GMM), particularly the Arellano-Bover/Blundel-Bond estimator. The results show a negative link between per capita income and CO_2_ emissions and fossil energy consumption. A positive relationship is found between per capita income and nitrous oxide. The study finds that the EKC model holds more in the case of nitrous oxide compared to CO_2_ emissions and fossil energy consumption which shows a U-shaped link in emerging economies.

Li et al. (2019) examine the spatial interactions of economic performance on carbon intensity of welfare in Chinese provinces for the period 1995–2016. The spatial model is used to test the presence of the EKC model. The findings reveal that there is an inverted N-shaped EKC model. Thus, GDP has two turning points. Ding et al. (2019) examine the presence of the EKC model of PM2.5 pollution in China (Beijing-Tianjin-Hebei region). The research applies spatial econometric modelling. The study finds that there exists an inverted U-shaped EKC regarding economic growth and PM2.5. Also, Wang and He (2019) use spatial dependency to test the EKC in China and find an N-shaped relationship. The study also reports that economic relationships significantly outperform the other relationships in measuring the spatial dependency between provinces.

Xie et al. (2019) apply different estimation techniques such as the spatial lag model, the spatial autoregressive model with spatial autoregressive disturbances, two-stage least squares regression, quantile regression, and nonparametric regression to test whether there is an EKC between economic growth and smog pollution in China. The results uncover that PM2.5 pollutants demonstrate strong positive characteristics of spatial spill-over. The results also indicate that there is a significant inverted U-shaped relationship between economic growth and PM2.5 concentrations, which confirms the EKC hypothesis.

Wang and He (2019) use a spatial econometric tool to examine to what extent CO_2_ spill-over depends on provinces’ bilateral geographical and economic linkages. The results reveal an N-shaped relationship, either before or after grouping, which demonstrate a strong correlation against the effectiveness of the EKC hypothesis. The results show that even though considering the spatial aggregation effect, the economic relations behind CO_2_ emissions can also capture the spatial characteristics more accurately, providing valuable references for policymaking and carbon emission reduction.

Ding et al. (2019) utilise satellite observations of PM2.5 pollution and panel data of 13 cities in the Beijing–Tianjin–Hebei region over the period 1998 to 2016 to analyse the relationship between economic growth and PM2.5 pollution. After verifying the spatial effect of PM2.5 pollution by spatial statistical analysis, the spatial Durbin model (SDM) was chosen over the non-spatial model to examine the EKC hypothesis. The results demonstrate that the relationship between economic growth and PM2.5 pollution follows an inverted U-shaped pattern. Though the Beijing–Tianjin–Hebei region is still in the upward section, it is likely to cross the turning point and enter the downward section in the future. Second, the turning points of EKC would happen later with spatial effect considered, compared to the result of the non-spatial model. Third, the turning point of EKC would not happen until the region enters the post-industrial economy stage.

A study by Mahmood (2020a) examines the determinants of CO_2_ emissions per capita by utilising spatial econometric modelling using a group of 21 North American nations. The study finds evidence of spatial dependence in per capita carbon dioxide emissions and its explanatory factors. The study also finds negative spill-over effects for all hypothesised explanatory variables, but per capita income has a positive impact. There exists the presence of the EKC, and the turning point is estimated at USD 15,665 per capita income. Six countries are found to be on the second stage of the inverted U-shaped EKC.

Fong et al. (2020) present the first spatial econometric assessment of the EKC for Southeast Asia. The income–pollution trajectories of three air pollutants—nitrogen oxides, sulphur dioxide, and PM2.5—are examined through standard and spatial EKC models that regress per capita emissions on several socioeconomic indicators. The econometric assessment utilises data over the period 1993 to 2012 for nine Southeast Asian countries at varying levels of economic development.  The results demonstrate an inverted U-shaped curve for all pollutants, therefore confirming the existence of an EKC. Spatial spill-overs are not found for nitrogen oxide emissions but are supported for SO2 and PM2.5 emissions.

Chang et al. (2021) investigate the relationship between air pollution and economic growth using an augmented green Solow model. Using data on 284 cities in China over the period 2004 to 2015, the study investigates the EKC hypothesis using spatial dynamic panel data models. The model results shows an inverted U-shaped EKC, and indicate that the peak will come earlier due to the spill-over effect of abatement technology. The shape of the EKC is backed by empirical evidence and is robust when cities are grouped by income level, region, and secondary industry. The results also demonstrate that the shape of the EKC is sensitive to the model specification of dynamic and spatial charactersitics. More recently, using the spatial Durbin model, Mahmood (2023) investigates the effects of foreign direct investment, exports, and imports on emissions in 18 Latin American countries over the period 1970 to 2019, using spatial econometric analysis. The EKC is validated in the study.

In the spatial analysis, it is evidenced from the literature that none exists in the context of SSA. The studies that were conducted in the African context focused on the conventional EKC model using static panel data econometric studies. However, little is known as to the applicability of the spatial EKC in these SSA countries. Thus, a distinctive element of this study is that it adds the spatial econometric modelling in the context of SSA countries to capture spatial interactions in the EKC framework.

## Methodology and data

### Theoretical framework

From the seminal work of Grossman and Krueger (1995), the baseline model to evaluate the EKC hypothesis is given as

1 $$ln{E}_{it}= {\beta }_{0}+{\beta }_{1}ln{Y}_{it}+ {\beta }_{2} ({ln{Y}_{it})}^{2}+ {\upvarepsilon }_{it}$$

where ${E}_{it}$ stands for the environmental degradation level in year *t*, for country *i*; ${Y}_{it}$ represents the output per capita level of country *i* in the year *t*; ${\beta }_{1}$ is a positive slope; ${\beta }_{2}$ is a negative slope; and ${\upvarepsilon }_{it}$ is the error. The log–log functional form is used to reduce data volatility and the potential existence of heteroscedasticity (Friedl and Getzner 2003).

Equation (1) is augmented to include ${\mathrm{z}}_{it}$ to have Eq. (2):

2 $$ln{E}_{it}= {\beta }_{0}+{\beta }_{1}ln{GDPPC}_{it}+ {\beta }_{2} ({ln{GDPPC}_{it})}^{2}+ \sum_{j}{n}_{j}{z}_{it}^{j}+ {\upvarepsilon }_{it}$$

In (2), the coefficients reflect the elastic characteristics. Two alternative indicators of environmental degradation level are used: carbon dioxide emission intensity level and natural resource depletion. As such, variable E can either represent CO_2_ emission (CO2E) or natural resource depletion (NATDEP). GDP per capita (GDPPC) is a proxy for ${Y}_{it}$.

In the model, ${z}_{it}^{j}$ is one of the control factors that influence the environmental degradation. This includes capital-to-labour ratio (CL), urban population as a percentage of total population (URBAN), energy intensity level of primary energy (ENERIN), net ODA received as a percentage of GNI (NODA), trade as a percentage of GDP (TOP), GDP per land area (GDPTLA), and Governance Index (GOV). The selection criteria of these control variables are based on the relevant literature (Hao et al. 2016; Shahbaz et al. 2016; Ogundari et al. 2017; Wang and Ye 2017; Hove and Tursoy 2019; Li et al. 2019; Demissew Beyene and Kotosz 2020; Mahmood, 2020a).

The expected signs for the main coefficients are as follows:

I) Environment Kuznets curve: ${\beta }_{1}>0$ and ${\beta }_{2}<0$

Initially, it is anticipated that higher per capita income increases environmental degradation. In the context of an economic agent facing the choice of two products, consumption good versus environmental quality, it is expected that as the individual satisfies its basic needs, the marginal utility of consumption will start to decrease and that of environmental quality will increase. As such, after reaching a turning point, there will be a negative growth rate in environmental degradation as per capita income rises.

II) Capital-to-labour ratio: ${\beta }_{3}>0$

The capita-to-labour ratio measures the composition effect. The composition effect can be negative or positive depending on the resource endowments of each country as well as the strength of environmental policy (Tsurumi and Managi 2010). Therefore, it is anticipated that countries in the SSA region are endowed with abundant natural resources and likely to relatively specialise in natural resources sectors which in turn worsen environmental problems.

III) Urbanisation: $${\beta }_{4}<0$$

Urbanisation is the term used to describe the physical expansion of urban areas brought on by suburbanisation and rural migration (Effiong 2016). Urbanisation growth is considered to be a significant component that raises citizen demand for a better environment and thus improves environmental quality. Reduced pollution due to economies of scale in the creation of adequate and effective public infrastructure is another way that urbanisation improves the environment (Effiong 2016). Urbanisation is therefore anticipated to enhance environmental quality in this situation.

IV) Energy intensity level of primary energy: ${\beta }_{5}>0$

Energy intensity of primary energy refers to the ratio between energy supply and GDP measured at the purchasing power parity. Thus, it measures how much energy is utilised in the production of one unit of economic output. A lower ratio indicates lower energy used to produce a unit of output. As a proxy for energy consumption, it can increase the amount of carbon emissions and hence increase environmental degradation (Munir & Ameer 2018).

XXII) Net ODA received as a percentage of GNI: ${\beta }_{6}<0$

This was included as a proxy to measure the impact of sustainable development assistance on environmental quality. It is expected that the funding for development flows from the developed countries to developing countries where there is a low willingness to pay for environmental quality (Pindiriri and Chidoko 2012). Thus, it is expected to enhance the quality of the environment, thus a negative coefficient.

VI) Trade (% of GDP): ${\beta }_{7}>0$

Trade in this case represents the trade openness which enters the model so as to measure the effects of trade liberalisation on environmental quality as well as the PH hypothesis. The PH hypothesis asserts that due to weak environmental regulations as countries open for more trade, developed countries tend to move their dirty industries to developing countries. Thus, for developing countries, there will be more environmental degradation. Therefore, trade openness is expected to be detrimental to environmental quality in SSA countries. Thus, the coefficient of trade openness is anticipated to be positive.

VII) GDP per land area: ${\beta }_{8}>0$

This variable measures the scale of economic activity (Antweiler et al. (2001). The GDP for every country is divided by that country’s total land area. It is preferred as a measure of scale of economic activity since it is measured in its intensive form and it captures variations in the flow of economic activity per unit area across nations which also vary in density and size (Antweiler et al. 2001). Thus, it is anticipated that a rise in the scale of economic activity worsens existing environmental problems.

VIII) Governance index: ${\beta }_{9}<0$

It is projected that better governance will play a significant role in accelerating the occurrence of the environmental Kuznets curve’s turning point.

### Data

This study collects balanced panel data for 35 countries in SSA covering the period 2000–2015 on all the selected variables, dictated by data availability, which provides 490 observations. Macro-level data is retrieved from the World Development Indicators (WDI) and World Governance Indicators (WGI). Table 1 shows the description of the variables, and Table 2 provides the descriptive statistics.

**Table 1:** Definition of the variables used (over the period 2000–2015)

Variables	Definition	Source
CO2E	CO_2_ emissions (kg per 2010 US$ of GDP)	WDI
NATDEP	Adjusted savings: natural resources depletion (% of GNI)	WDI
GDPPC	GDP per capita (constant 2010 US$)	WDI
CL	Capital-to-labour ratio	WDI
URBAN	Urban population (% of total population)	WDI
ENERIN	Energy intensity level of primary energy (MJ/$2011 PPP GDP)	WDI
NODA	Net ODA received as a percentage of GNI	WDI
TOP	Trade (% of GDP)	WDI
GDPTLA	GDP per land area (sq. km)	WDI
GOV	Governance index	WGI

**Table 2:** Summary statistics

Variables	Mean	Standard deviation	Minimum	Maximum
CO2E	0.291	0.206	0.064	1.471
NATDEP	6.601	9.15	0	51.599
GDPPC	1944.223	2358.021	221.096	9879.346
CL	1,732,469	2,880,934	88,039.61	18,000,000
URBAN	38.034	16.077	8.682	88.118
ENERIN	7.569	4.692	1.91	25.723
NODA	8.181	7.631	− 0.251	62.187
TOP	66.919	27.458	21.333	175.798
GDPTLA	218,197.2	779,090.8	2394.606	5,894,229
GOV	− 0.606	0.57	− 1.719	0.88

CO2E and NATDEP are used as proxies to capture environmental quality. The governance index is computed as the mean of the six dimensions of governance provided by the WGI of the World Bank: voice and accountability, political stability and absence of violence, government effectiveness, regulatory quality, rule of law, and control of corruption, to capture the quality of governance.

### Moran index

The study examines whether there is any regional association between the two environmental quality measures. The Moran index, which is given as follows, is frequently used to assess spatial correlation (Cliff and Ord 1970; Anselin 1995).

3 $$I= \frac{\sum_{i=1}^{n}\sum_{j=1}^{n}{W}_{ij}{y}_{i}{y}_{j}}{\sum_{i=1}^{n}{y}_{i}^{2}},$$

where *n* stands for the number of countries in the panel; ${\mathrm{y}}_{i}$ represents the value of environmental degradation for country *i* centred to the mean; and ${\mathrm{W}}_{ij}$ captures the ${ij}^{th}$ element of the spatial weight matrix *W*. *W* represents a symmetric *A* × *A* weight matrix in which each element is computed as 1/${\updelta }_{ij}$. ${\updelta }_{ij}$ measures the Euclidean distance between country *i* and country *j* (Dubin 1988).

The Moran index is the correlation coefficient between the value of the environmental degradation and its spatial lag. The Moran index ranges from − 1 to 1, with values above 0 depicting positive spatial correlation and values depicting negative spatial correlation amongst different countries. Moreover, when the value is closer to zero, it represents weaker spatial correlation (Jeetoo 2020).

### Spatial panel econometric model

As a starting point, the spatial autoregressive model (SAR) can be utilised to factor in spatial interaction effects in testing for the presence of the EKC in environmental quality (Jeetoo 2020, 2022a) and is given in the form below:

4 $${y}_{it}= {\mathrm{\rho Wy}}_{it}+ {x}_{it}\upbeta +{\upvarepsilon }_{it}$$

where ${x}_{it}$ = (${x}_{it,1}$,…, ${x}_{it,H}$). The *H* variables (which excludes the linear time trend) are in logarithmic format and thus the coefficients *β* = (${\beta }_{1}$,…, ${\beta }_{K}$) can be interpreted as elasticities, for countries *i* in year *t*. The error terms are given as ${\upvarepsilon }_{it}$, where ${\upvarepsilon }_{it}= {\mathrm{\alpha }}_{i} + {\mathrm{v}}_{it}$. ${\mathrm{\rho Wy}}_{it}$ is an endogenous interaction. *W* represents a symmetrical spatial weight matrix G × G (which is non-stochastic) where the element *ωij* is given as 1/δ*ij*. δ*ij* measures the distance between country *i* and country *j* (where *i* ≠ *j*) (Dubin 1988; Garrett et al. 2007).

A second way to model spatial interaction is to focus on the error process, referred to as the spatial error model (SEM). Under the latter, given unobserved common conditions of close countries in a region, spatial dependence is modelled as

5 $${y}_{it}= {x}_{it}\upbeta +{\mathrm{\alpha }}_{i} + {\mathrm{u}}_{it}$$

where ${\mathrm{u}}_{it}=\mathrm{\lambda W}{\mathrm{u}}_{it}+{\mathrm{z}}_{it}$ for independent errors ${\mathrm{z}}_{it}$(Jeetoo 2020, 2022a).

The spatial Durbin model (SDM) is another way to model spatial dependence, and it is a nesting extension of both the SAR and SEM models. It is expressed as

6 $${y}_{it}= {\mathrm{\rho Wy}}_{it}+ {x}_{it}\upbeta +\mathrm{W}{x}_{it}\uptheta +{\upvarepsilon }_{it}$$

Equation (6) includes both the spatially lagged dependent variable and the spatially autocorrelated errors.

To start with, this study chooses the SDM model for the analysis. A likelihood ratio method based on the SDM can help with the problem of discriminating between the SAR and SEM models, which are non-nested alternatives. Additionally, it is possible to think of the SDM as a weighted average of the SAR and SEM models (Jeetoo 2020, 2022a, b).

## Empirical results and discussion

### Moran’s I

Table 3 includes estimates for the Moran index and results for the regional association of environmental quality across different nations. The results indicate positive spatial correlation for the whole period under consideration for both variables lnCO2E and lnNATDEP; this provides evidence of spatial agglomeration. In addition, it is observed that the spatial correlation in lnCO2E has strengthened over the corresponding period of the study.

**Table 3:** Spatial correlation test of REC

Year	lnCO2E		lnNATDEP	
	Moran’s I value	*p*-value	Moran’s I value	*p*-value
2002	0.14408	0.05660	0.20098	0.01241
2003	0.20542	0.00929	0.18743	0.01887
2004	0.17967	0.02046	0.19168	0.01666
2005	0.19495	0.01370	0.19986	0.01318
2006	0.23451	0.00364	0.18682	0.01947
2007	0.26669	0.00110	0.18298	0.02198
2008	0.31089	0.00017	0.18531	0.02077
2009	0.29350	0.00033	0.20681	0.01088
2010	0.28822	0.00047	0.21246	0.00904
2011	0.23256	0.00383	0.21553	0.00830
2012	0.29451	0.00035	0.18117	0.02334
2013	0.23054	0.00381	0.17614	0.02736
2014	0.23326	0.00384	0.20273	0.01304
2015	0.25337	0.00177	0.17056	0.03257

### Spatial econometric model analysis

After testing for spatial correlation in environmental quality (using the two proxies) between countries in SSA, this study utilises various spatial panel econometric models to investigate the effect of GDP per capita, capital-to-labour ratio, urbanisation, energy intensity, net official development assistance, trade openness, GDP per land area, and democracy level on environmental quality. Using both proxies (CO2E and NARDEP), four classical panel models are estimated: pooled OLS individual fixed effects, time effects or two-way effects (Elhorst 2014).

Table 4 displays the findings based on the four traditional panel models estimated for CO2E: CM1.pooled-ols (pooled), CM2.individual-FE (individual fixed effects), and CM3.two-way-FE (OLS with both individual and time fixed effects). The standard error provided is computed using the Driscoll and Kraay (1998) method because of the presence of autocorrelation, heteroskedasticity, and cross-sectional dependence in the dataset. Note that the fixed effect (FE) model was selected based on the Hausman test with test statistic chi^2^(9) = 62.42 with a *p*-value of 0.000, which indicates rejection of the random effect specification.

**Table 4:** Traditional panel models—CO2E

	CM1.pooled-ols (pooled)	CM2.individual-FE (individual fixed effects)	CM3.two-way-FE (both individual and time fixed effects)
lnGDPPC	0.175	T-1.098*	− 3.518***
	(0.315)	(0.586)	(0.784)
lnGDPPC2	− 0.044**	0.002	0.103**
	(0.019)	(0.035)	(0.041)
lnCL	0.268***	0.421***	0.415***
	(0.056)	(0.086)	(0.091)
lnURBAN	0.543***	1.070***	1.098***
	(0.047)	(0.227)	(0.224)
lnENERIN	0.434***	0.268***	0.311***
	(0.038)	(0.062)	(0.061)
lnNODA	− 0.222***	− 0.040*	− 0.005
	(0.036)	(0.021)	(0.022)
lnTOP	0.096**	0.078*	0.097**
	(0.048)	(0.045)	(0.046)
lnGDPTLA	0.071***	0.356***	1.589***
	(0.013)	(0.134)	(0.292)
lnDEM	1.407***	− 0.006	− 0.100
	(0.101)	(0.153)	(0.155)
Constant	− 8.989***	− 7.823***	− 9.139***
	(1.102)	(2.031)	− 2.024)
AIC	344.662	− 522.591	− 528.647

Akaike information criterion (AIC) is utilised to choose whether individual-FE or two-way-FE is the most appropriate (Akaike 1974). The model with the lowest AIC indicator is chosen, i.e., the two-way-FE model (CM3.two-way-FE) in this case.

Table 5 provides the results estimated with the four traditional panel models for NATDEP: NM1.pooled-ols (pooled), NM2.individual-RE (individual fixed effects), and NM3.two-way-RE (OLS with both individual and time fixed effects). Again, the standard error is based on the Driscoll and Kraay (1998) methodology. Note that the random effect (RE) model was chosen based on the Hausman test with test statistic chi^2^(9) = 13.68 with a *p*-value of 0.134, which shows that random effect specification is chosen. Moreover, the two-way RE model was selected over the one-way individual RE model, though their results are almost similar.

**Table 5:** Traditional panel models—NATDEP

	NM1.pooled-ols (pooled)	NM2.individual-RE (individual effects)	NM3.two-way-RE (both individual and time fixed effects)
lnGDPPC	− 2.242***	5.026***	4.546***
	(0.847)	(1.062)	(1.019)
lnGDPPC2	0.124**	− 0.253***	− 0.246***
	(0.050)	(0.068)	(0.065)
lnCL	0.530***	− 0.648***	− 0.336**
	(0.151)	(0.168)	(0.168)
lnURBAN	− 0.423***	− 0.359	− 0.382
	(0.125)	(0.284)	(0.294)
lnENERIN	− 0.200*	0.259**	0.237**
	(0.103)	(0.124)	(0.118)
lnNODA	0.030	0.055	0.056
	(0.097)	(0.045)	(0.043)
lnTOP	0.252*	0.497***	0.363***
	(0.130)	(0.096)	(0.093)
lnGDPTLA	0.014	0.056	0.029
	(0.035)	(0.097)	(0.110)
lnDEM	− 2.643***	− 2.031***	− 1.788***
	(0.272)	(0.318)	(0.307)
Constant	7.225**	− 12.200***	− 12.690***
	(2.964)	(3.734)	(3.601)

After selecting the two-way-fixed effect formulation for CO2E and the two-way-random effect formulation for NATDEP, the SAR, SEM, and SDM models are estimated for both proxies, i.e., CO2E and NATDEP. Amongst the three functional forms, the selected specification is the one with superiority based on the following criteria: number of significant coefficients and level of *R*-squared (Amidi et al. 2020).

For CO2E, Table 6 provides the estimations for the SAR, SEM, and SDM models. All the models have 8 significant coefficients, except for the SDM model which has only 7 significant coefficients. Moreover, the SAR has the highest *R*-squared value. Therefore, the model SAR was chosen for analysis on the factors affecting CO2E in SSA.

**Table 6:** Determinants of CO2E models

	Two-way SAR(FE)	Two-way SEM(FE)	Two-way SDM(FE)
$\rho$	− 0.242***		− 0.321***
	(0.074)		(0.085)
$\lambda$		− 0.306***	
		(0.082)	
lnGDPPC	− 3.375***	− 3.372***	− 3.621***
	(0.729)	(0.723)	(0.752)
lnGDPPC2	0.093**	0.094**	0.120***
	(0.038)	(0.038)	(0.041)
lnCL	0.386***	0.391***	0.472***
	(0.085)	(0.083)	(0.096)
lnURBAN	1.050***	1.099***	0.909***
	(0.208)	(0.209)	(0.229)
lnENERIN	0.321***	0.327***	0.337***
	(0.057)	(0.057)	(0.064)
lnNODA	− 0.004	0.001	0.008
	(0.020)	(0.020)	(0.020)
lnTOP	0.095**	0.105**	0.072*
	(0.043)	(0.042)	(0.044)
lnGDPTLA	1.618***	1.617***	1.520***
	(0.271)	(0.268)	(0.278)
lnDEM	− 0.076	− 0.066	− 0.232
	(0.144)	(0.140)	(0.161)
W.lnGDPPC			3.321
			(2.663)
W.lnGDPPC2			− 0.236
			(0.171)
W.lnCL			− 0.004
			(0.276)
W.lnURBAN			0.705
			(0.968)
W.lnENERIN			0.258
			(0.232)
W.lnNODA			0.088
			(0.064)
W.lnTOP			− 0.004
			(0.122)
W.lnGDPTLA			0.038
			(0.485)
W.lnDEM			0.275
			(0.418)
*R*-squared	0.044	0.038	0.004

For NATDEP, Table 7 provides the estimations for the SAR, SEM, and SDM models. The SAR and SDM models have 10 significant coefficients, while the SEM has only 8 significant variables. The SDM has the highest *R*-squared value. Therefore, the model SDM is chosen for analysis on the determinants of NATDEP in SSA.

**Table 7:** Determinants of NATDEP models

	Two-way SAR(RE)	Two-way SEM(RE)	Two-way SDM(RE)
$\rho$	0.275***		0.148***
	(0.034)		(0.055)
$\lambda$		0.281***	
		(0.038)	
lnGDPPC	4.324***	4.441***	3.965***
	(0.995)	(1.013)	(1.017)
lnGDPPC2	− 0.222***	− 0.238***	− 0.199***
	(0.063)	(0.065)	(0.065)
lnCL	− 0.284*	− 0.279	− 0.101
	(0.162)	(0.172)	(0.167)
lnURBAN	− 0.722***	− 0.531*	− 0.947***
	(0.261)	(0.296)	(0.331)
lnENERIN	0.209*	0.201*	0.172
	(0.116)	(0.117)	(0.116)
lnNODA	0.0483	0.050	0.039
	(0.042)	(0.042)	(0.042)
lnTOP	0.390***	0.366***	0.353***
	(0.090)	(0.094)	(0.095)
lnGDPTLA	− 0.068	0.002	− 0.163
	(0.087)	(0.109)	(0.105)
lnDEM	− 1.803***	− 1.689***	− 1.733***
	(0.296)	(0.310)	(0.302)
W.lnGDPPC			2.562**
			(1.112)
W.lnGDPPC2			− 0.083
			(0.072)
W.lnCL			− 0.824***
			(0.313)
W.lnURBAN			− 0.492
			(0.487)
W.lnENERIN			0.182
			(0.340)
W.lnNODA			0.039
			(0.114)
W.lnTOP			0.225
			(0.200)
W.lnGDPTLA			− 0.004
			(0.231)
W.lnDEM			− 1.317**
			(0.632)
Constant	− 11.64***	− 12.31***	− 12.040***
	(3.493)	(3.700)	(3.820)
*R*-squared	0.210	0.064	0.416

The coefficient in Table 7 is positive. This demonstrates a positive spatial spill-over impact of a country’s NATDEP on its neighbours. Based on Moran’s I, this shows that NATDEP in Africa has spatial agglomeration. Since the coefficient of spatial autocorrelation is much higher than zero, NATDEP in Africa displays characteristics of high-high agglomeration and low-low agglomeration. This shows that increased (decreased) NATDEP in a country and vice versa are caused by greater (lower) NATDEP levels in the country’s neighbours. The significant linkages, such as geographic proximity and bilateral economic ties as far as the usage of natural resources is concerned, provide an explanation for the transmission mechanism. This implies that in Africa, there is a tendency for close countries to have the same policies regarding natural resources exploitation. As such, if a country of close to another one which is exploiting its natural resources, the country will also tend to follow the same trend. This could be due to the fact that close countries are also endowed with similar resources, and they tend to mimic the behaviour of their neighbours regarding natural resources exploitation.

Spill-overs in NATDEP can emerge due to emulation behaviour by governments, partly as a result of the phenomenon known as “yardstick competition” (Revelli 2006). Countries can “emulate” their neighbouring governments with regard to their level of natural resources exploitation. Based on the yardstick competition theory (Revelli 2006), it can be argued that governments have the tendency to match the way of behaving of close countries as voters often make comparisons with neighbouring countries. Moreover, the “demonstrative effect” (Moscone et al. 2007) can be used to explain spatial spill-over in public healthcare expenditure patterns. Some countries may exercise leadership in applying policies in increasing natural resources exploitation giving rise to the “following effect” in other countries.

The CO2E coefficient, according to Table 6, is, nevertheless, negative. This can be understood as a negative spatial spill-over effect of a country’s CO2E on that of its neighbours. This indicates that increased (decreased) CO2E in a country is caused by lower (greater) CO2E levels in the country’s neighbours. This is in contrary to the findings regarding NATDEP. Therefore, there is strong evidence that countries in Africa do not follow each other regarding CO2E, given the absence of spatial interdependence. Actually, it seems that African countries have opposite CO2E compared to their neigbouring countries. This could be explained by the fact that certain countries have more production due to the fact that they attract higher FDI at the detriment of neigbouring countries. As such, the neigbouring countries have lower production level and thus generate lower CO2E.

Regarding the effect of the explanatory variables, the conventional interpretation for coefficient estimation, as partial derivatives, is not possible in spatial models (Jeetoo 2020, 2022a). In the SDM model, the effect of each explanatory variable is decomposed into direct effect, indirect effects, and total effects (Anselin 1988; LeSage and Pace 2010; Jeetoo 2022a, b). Table 8 provides the spatial effect decomposition result.

**Table 8:** Spatial spill-over effect decom*position with SDM model for CO2E and NATDEP

Indicator name	CO2E – SDM(FE)			NATDEP – SDM(RE)		
	Direct effect	Indirect effect	Total effect	Direct effect	Indirect effect	Total effect
lnGDPPC	− 3.394***	1.040***	− 2.354***	4.168***	8.590**	12.760***
	(0.755)	(0.321)	(0.606)	(1.066)	(3.468)	(3.877)
lnGDPPC2	0.093**	− 0.028**	0.065**	− 0.207***	− 0.300	− 0.508**
	(0.034)	(0.013)	(0.030)	(0.067)	(0.212)	(0.235)
lnCL	0.400***	− 0.123***	0.277***	− 0.128	− 2.282***	− 2.410***
	(0.082)	(0.038)	(0.065)	(0.162)	(0.839)	(0.863)
lnURBAN	1.065***	− 0.329***	0.736***	− 0.991***	− 1.706	− 2.697*
	(0.203)	(0.101)	(0.158)	(0.310)	(1.450)	(1.480)
lnENERIN	0.325***	− 0.101***	0.225***	0.186	0.631	0.817
	(0.057)	(0.031)	(0.043)	(0.121)	(0.949)	(0.987)
lnNODA	− 0.003	0.001	− 0.002	0.045	0.147	0.192
	(0.020)	(0.006)	(0.014)	(0.045)	(0.339)	(0.361)
lnTOP	0.095**	− 0.029*	0.066**	0.363***	0.784*	1.147**
	(0.044)	(0.016)	(0.031)	(0.099)	(0.476)	(0.503)
lnGDPTLA	1.632***	− 0.503***	1.129***	− 0.169*	− 0.101	− 0.269
	(0.267)	(0.144)	(0.220)	(0.097)	(0.652)	(0.630)
lnDEM	− 0.064	0.019	− 0.046	-1.792***	− 4.295**	− 6.087***
	(0.144)	(0.045)	(0.100)	(0.295)	(1.851)	(1.879)
Turning point (USD)	89,197,593	108,834,406	81,802,163	23,568	1,650,628	284,661

The results show that the coefficients from the spatial models differ from those from the non-spatial models. The primary cause of these discrepancies is the neglect of the spatial spill-over impact of data. Another cause is the feedback effects that emerge from CO_2_ emissions or the depletion of natural resources in one country, which in turn affects the CO_2_ emissions or depletion of natural resources in neighbouring nations. Both the geographically lagged independent variables and the spatially lagged dependent variables contribute to the feedback effects in different ways (Kang et al. 2016).

Table 8 also presents the estimated EKC turning points. For the direct effects, the turning points are estimated by considering the national income elasticity using the formula: ${{GDP}^{*}}_{D}= {e}^{-\frac{{\uptheta }_{1}}{{2\uptheta }_{2}}}$). The indirect turning point is measured by considering the changes in neighbouring per capita income and is computed using the formula ${{GDP}^{*}}_{I}= {e}^{-\frac{{\uptheta }_{1}}{{2\uptheta }_{2}}}$. The total turning point, which is the sum of local and neighbouring changes, is calculated using the formula: ${{GDP}^{*}}_{T}= {e}^{-\frac{{(\beta }_{1}+ {\uptheta }_{1})}{{(2\beta }_{2}{+\uptheta }_{1})}})$.

With respect to the first model with CO_2_ emissions as a dependent variable, for the direct effects, a U-shaped relationship is noted between growth and environmental quality, which is the opposite of the EKC model. For the total effect, a U-shaped relationship is also uncovered between economic growth and CO_2_ emission. Therefore, referring to the direct and total effects, as income is increasing beyond the turning point, individual countries tend to become less concerned with the environmental quality. Shahbaz et al. (2016) also found similar results in the context of Sudan and Tanzania where there is a U-shaped relationship between economic growth and CO_2_ emissions. However, regarding the indirect effects, an inverted U-shape relationship is observed. This implies that there is some evidence of the EKC effect in relation to neighbouring changes in economic growth on a country’s CO2 emission. On the whole, economic growth shows a positive pressure on the home countries’ environment but unpleasant spill-overs in the neighbouring countries.

The negative total effect of economic growth on CO_2_ emissions as per findings of the spatial analysis conveys that economic growth is beneficial for the overall environment of Africa. The direct effect is − 3.394, the spill-over effect is 1.040, and the total effect in the whole region is − 2.354. Thus, the unpleasant environmental spill-overs in neighbouring countries are reducing the overall positivity of economic growth on CO2E in the whole (− 3.394 + 1.040 =  − 2.354).

In the case of model 2 with natural resources depletion as a dependent variable, there is the presence of the EKC when considering the direct effects where both the coefficients of GDP per capita and its square are statistically significant and in line with expectations, but the coefficient of the quadratic terms for the neighbouring effects is not statistically significant. The direct turning point income is computed to be USD 23,568. Thus, the relationship between the scale effect and technique effect with respect to GDP per capita and natural resources depletion is inverted U-shaped. This validates the existence of the EKC in the SSA countries in terms of natural resources depletion. Therefore, as income increases, initially natural resources depletion increases until the GDP per capita is at US$23,568. However, beyond this turning point, the positive effect of income is expected to turn into the negative which results in reduced natural resources depletion. Studies by Balado-Naves et al. (2018) and by Hove and Tursoy (2019) also find the presence of the EKC.

It is noted, from the sample used for this study, that the maximum per capita income (based on 2010 constant USD) is USD 9879. As such, none of the countries currently have reached the turning point USD 23,568. Therefore, it is anticipated that once the latter is reached, countries will start to reduce the natural resource depletion rate. This could be due to the fact that after a certain threshold of depletion, countries start to realise the severity of the depletion situation. Moreover, the coefficient of the indirect effects is positive. This shows that the effect of growth in neigbouring countries reinforces the depletion of natural resources in a country, and therefore, the over impact is higher (4.168 + 8.59 = 12.75).

As expected, the coefficient of the capital-to-labour ratio (CL) is positive and statistically significant. Thus, if the capital-labour ratio increases by 1%, CO_2_ emissions also increase by 0.4%. This supports the fact that most SSA countries are abundant in natural resources; hence, they produce goods which are relatively intensive in natural resources, such as mining and agriculture. As a result, they experience environmental degradation in the form of increased CO_2_ emissions. Furthermore, the result indicates a negative and statistically significant negative impact of the CL ratio of neighbouring countries on a country’s level of CO_2_ emissions as well as on a country’s natural resources depletion rate. This is interpreted as an increase in CL ratio in neighbouring countries reduces environmental degradation in a country. This can be because when neighbouring countries invest more in capital to extract and use resources, they deplete their level of natural resources and produce more CO_2,_ and at the same time, this reduces the level of natural resource usage in a particular country, and therefore, the latter faces less environmental degradation. Using non-spatial modelling, Nkengfack et al. (2019) also found that capital-labour ratio increases carbon dioxide emissions in SSA countries.

The urbanisation coefficient is statistically significant and positively influences CO_2_ emissions. Thus, a 1% increase in urbanisation increases CO_2_ emissions by 1.065%. Thus, the more people are increasing in the urban areas, the more they are increasing emissions. Also, urbanisation implies increases in industrialisation as some of the industries are in urban areas; this, in turn, also contributes to increased emissions. This finding aligns with Ma et al. (2016) and Wu et al. (2018), who concluded that accelerating urbanisation promoted population growth and energy use while causing many sources of CO_2_ emissions. Zhang and Lin (2012) and Kang et al. (2016) also find similar evidences. However, an increase in urbanisation in neighbouring countries leads to less CO_2_ emission in a country. Regarding natural resources depletion, there is a negative relationship between urbanisation and natural resources depletion. A 1% increase in urbanisation decreases natural resources depletion by 0.991%. Effiong (2016) also finds a negative relationship between urbanisation and environmental degradation.

Energy intensity has a positive influence on CO_2_ emissions only. Thus, a 1% increase in energy use intensity increases CO_2_ emissions by 0.325%. Thus, increased energy use is detrimental to the environment which is similar to the findings of previous studies (Cole and Neumayer 2004; Poumanyvong and Kaneko 2010; Du et al. 2012; Liu et al. 2015; Balado-Naves et al. 2018; Shahbaz et al. 2016) also found that energy consumption is detrimental to environmental quality. This shows the importance of energy efficiency in reducing CO_2_ emissions. However, the results also show a negative impact of the energy intensity of neighbouring countries on a country’ CO_2_ emissions.

Trade openness is detrimental to environmental quality on both CO_2_ emissions and natural resources depletion. The coefficients of trade openness in both models are statistically significant. A 1% increase in trade openness increases CO_2_ emissions by 0.09% and increases natural resources depletion by 0.36%. This finding relates to the theoretical model of Suri and Chapman (1998) that exports have a scale effect on a country and would increase the energy demand and pollution. This supports the pollution haven hypothesis, according to which high-income countries export their pollution to low-income countries by importing goods from low-income countries, and the positive impact of trade openness thus demonstrates that the developing countries in the SSA region are pollution havens. Thus, the industrialised world is able to shift its polluting businesses to poor nations due to the lax environmental standards. Also, these weak environmental regulations contribute to natural resources depletion as trade increases. Thus, trade liberalisation is detrimental to environmental quality in SSA.

By promoting commercial activity like mining rather than the importation of energy-efficient technologies that could minimise CO_2_ emissions, trade openness worsens environmental damage (Adu and Denkyirah 2017). Akin (2014), Iwata et al. (2012), Sharma (2011), Adu and Denkyirah (2017), and Ogundari et al. (2017) are just a few of the studies that show that trade openness causes more environmental degradation in developing nations. However, other authors found the opposite in China (Dong et al. 2010; Kang et al. 2016).

The variable which measures the scale of economic activity (i.e., GDP per land area) is positive and statistically significant on CO_2_ emissions. This supports the argument that a rise in the scale of economic activity worsens CO_2_ emissions. However, a higher scale of economic activity is found to reduce natural resource depletion in SSA. This could be because of increasing economic activities, which are mainly concentrated in manufacturing sectors, that in turn reduces the rate at which natural resources are depleting. The coefficient of democracy level (DEM), which is a measure of good governance, is negative and statistically significant on natural resources depletion, both in terms of direct effect and indirect effects. This indicates that better governance reduces natural resources depletion not only in a country but also has positive spill-over effects on neighbouring countries also.

## Conclusion and policy implications

The study’s conclusions addressing the depletion of natural resources in SSA are consistent with the EKC theory. Regarding CO_2_ emission, the EKC hypothesis is not supported. In terms of the depletion of natural resources, this supports the existence of the EKC in the SSA countries. Therefore, until the GDP per capita reaches US$ 23,568, natural resource depletion first increases as income increases. Beyond this tipping point, however, the positive impact of income is anticipated to flip into the negative, which results in a reduction in the depletion of natural resources. It should be mentioned that the maximum per capita income for the sample utilised for this study is USD 9879 (calculated using constant 2010 USD). As a result, none of the nations have yet to reach the tipping point of USD 23,568. Therefore, it is projected that countries will start to slow down the rate of natural resource depletion once the latter is achieved. Therefore, it is crucial that the government develop regulations right once to prevent the depletion of natural resources and simultaneously encourage the use of renewable resources. Given the presence of spatial interdependence in both CO2E and natural resources depletion, policies should come at the regional level rather than the national level. In this context, spatially coordinated multi-scale policies are likely to enhance the effectiveness of mitigation and adaptation strategies amidst environmental degradation in view of promoting sustainable development. This must be incorporated in the agenda of regional blocs such as the African Union and the SADC.

The findings offer useful information on economic variables that may have an effect on the environment in Africa nations and inform the formulation of policies aimed at enhancing the region’s ecological profile. CO_2_ emissions and the depletion of natural resources both show statistically significant cross-border spill-over effects. In order to combat the depletion of natural resources and CO_2_ emissions, governments should promote regional cooperation. Collaboration in the development of green energy, particularly in areas like clean development practices and carbon exchange trading legislation, needs to receive more attention. Authorities should establish economic-related policies and environmental regulations in order to increase environmental protection from bilateral economic and trade exchanges. Given the spatial dimension in environmental degradation in Africa, regional policies would be more relevant; these are anticipated to affect the turning point of the EKC for natural resources depletion within this region in which the initial trade-off between economic growth and environmental degradation is anticipated to be reduced at a faster rate and at a comparatively lower level of national income.

Regarding CO_2_ emissions and the depletion of natural resources, trade openness has a detrimental effect on the environment. The benefits of trade openness show that the developing nations in the SSA region are pollution havens because the findings are consistent with the pollution haven hypothesis, which claims that high-income countries export their pollution to low-income countries by importing goods from low-income countries. As a result, the developed world is able to move its polluting industries to poor nations due to the lax environmental standards. Additionally, as commerce grows, these lax environmental rules lead to the loss of natural resources. In order to prevent the developed world from moving its polluting businesses to SSA countries, more strict environmental legislation and regulations must be created.

Economic growth is shown to have detrimental effects, both in terms of direct effects and cross-country spill-over effects. Therefore, there is a need to check economic and trading activities to be responsible and sustainable as per SDGs. At the country or regional level, selective taxation could be imposed on economic and trade activities which causes harm in terms of CO_2_ emissions and natural resources depletion. The funds raised can be used to subsidise economic and trade activities in industries which uses renewable sources of energy which produces less pollution and which does not negatively impact natural resources depletion. Furthermore, at the national and regional level, more should be invested to promote research and development activities to produce less environmentally harmful technologies, which could reduce environmental degradation. In addition, authorities should conduct economic-related strategies and environmental regulations to increase environmental protection from bilateral economic and trade exchanges.

The study demonstrates that greater CO_2_ emissions result from a country’s capital-to-labour ratio. The study discovers an inverse association between capital-to-labour ratio in neighbouring nations and environmental degradation in a country because a rise in capital-to-labour ratio in neighbouring countries reduces both CO_2_ emissions and natural resource depletion in a country. The findings also show that while urbanisation has a good effect on a nation’s CO_2_ emissions, it has a detrimental effect on the depletion of natural resources. It is interesting to note that urbanisation in nearby nations has a detrimental impact on a nation’s CO_2_ emissions as well.

Keeping the detrimental effects of urbanisation on CO_2_ emissions and natural resource depletion within the African economies into consideration, it is recommended that the governments and regional bodies pay a particular attention to unplanned urbanisation issues that could potentially contribute to higher CO_2_ emission and natural resource depletion within the concerned economies. In addition, the negative effects of urbanisation on the environment can also be dealt by promoting the use of renewable energy within the urban industrial sectors which could reduce the intensity of CO_2_ emission without demeaning the potential for sustained industrialisation across African countries. As such, such policies should be carefully thought and planned to avoid the potential pitfalls associated with it, which can have even more harmful detrimental effects for the region.

Energy intensity has a positive influence on CO_2_ emissions, but the results also show a negative impact of energy intensity of neighbouring countries on a country’ CO2 emissions. The scale of economic activity also has a positive impact on CO_2_ emission, but it has a negative effect on natural resource depletion in SSA. Given the foregoing, it is essential that policymakers develop an integrated strategy with the aim of lowering CO_2_ emissions and natural resource depletion based on the factors. Building energy-efficient, low-carbon, and renewable urban infrastructure is crucial for sustainable development. For instance, regional blocs should promote renewable energy development through incentives for renewables, provision of tax credit to renewable generators, and provision of subsidies for infrastructure installation for renewable energy generation. During the processes of capital investment, urbanisation, and expanding scale of activity, the aforementioned should be encouraged. Additionally, SSA nations must seek efficiency-improving policies and interventions to stop environmental degradation (Adu and Denkyirah 2017).

The study also shows that better governance reduces natural resources depletion not only in a country but also has positive spill-over effects on neighbouring countries. The promotion of democratic principles and institutions, popular participation, and good governance is another objective of the African Union (2000). Similarly, the SADC Common Agenda is underpinned by the promotion, consolidation, and maintenance of democracy (SADC 1992). It is important for regional blocs together with individual countries in Africa to work together to promote good governance which should eliminate issues that negatively affect voice and accountability, political stability, government effectiveness, regulatory quality, rule of law, and control of corruption. This can reduce the problem of environmental degradation in the SSA region.

## Limitations of the study and future recommendations

The main limitation of the study emanates from the lack of data in the SSA region. Certain variables and countries could not be included as the model required a balance panel dataset to be able to estimate spatial effects. Moreover, the model did not capture dynamic effects in the spatial models.

Future research can extend this study to replicate the empirical investigation in the context of other group of countries for comparability and robustness check of the findings. Future research may also explore the application of dynamic spatial models. In addition, with more availability of data in the future, more relevant variables and countries in the SSA region can be added to the model.

## Data Availability

Data and materials are available upon request to the corresponding author.

## Abbreviations

AIC: Akaike information criterion
ARDL: Autoregressive distributed lag
CL: Capital-to-labour ratio
CO_2_: Carbon dioxide
CO2E: CO_2_ emissions (kg per 2010 US$ of GDP)
EKC: Environment Kuznets curve
ENERIN: Energy intensity level of primary energy (MJ/$2011 PPP GDP)
FE: Fixed effect
FGLS: Feasible generalized least square
FMOLS: Full modified ordinary least square
GDP: Gross domestic product
GDPPC: GDP per capita (constant 2010 US$)
GDPTLA: GDP per land area (sq. km)
GHG: Greenhouse gas
GMM: Generalized Means Model
GOV: Governance index
NATDEP: Adjusted savings: natural resources depletion (% of GNI)
NODA: Net ODA received as a percentage of GNI
PH: Pollution haven
PHH: Pollution haven hypothesis
PMG: Pooled mean group
RE: Random effect
SDM: Spatial Durbin model
SAR: Spatial autoregressive model
SDGs: Sustainable Development Goals
SEM: Spatial error model
SSA: Sub-Saharan Africa
TOP: Trade (% of GDP)
URBAN: Urban population (% of total population)
